# Spectroscopy and SEM imaging reveal endosymbiont-dependent components changes in germinating kernel through direct and indirect coleorhiza-fungus interactions under stress

**DOI:** 10.1038/s41598-018-36621-8

**Published:** 2019-02-07

**Authors:** Vladimir Vujanovic, Seon Hwa Kim, Rachid Lahlali, Chithra Karunakaran

**Affiliations:** 1 Department of Food and Bioproduct Sciences, University of Saskatchewan, 51 Campus Drive, Saskatoon, SK S7N 5A8 Canada; 2 Canadian Light Source, 44 Innovation Blvd, Saskatoon, SK S7N 2V3 Canada; 3 Present Address: Department of Crop Protection, Phytopathology Unit, Ecole Nationale d’Agriculture de Meknès, BP/S 40, Meknès, 50001 Morocco

## Abstract

In the present study, FTIR spectroscopy and hyperspectral imaging was introduced as a non-destructive, sensitive-reliable tool for assessing the tripartite kernel-fungal endophyte environment interaction. Composition of coleorhizae of *Triticum durum* was studied under ambient and drought stress conditions. The OH-stretch IR absorption spectrum suggests that the water-deficit was possibly improved or moderated by kernel’s endophytic partner. The OH-stretch frequency pattern coincides with other (growth and stress) related molecular changes. Analysis of lipid (3100–2800 cm^−1^) and protein (1700–1550 cm^−1^) regions seems to demonstrate that drought has a positive impact on lipids. The fungal endosymbiont direct contact with kernel during germination had highest effect on both lipid and protein (Amide I and II) groups, indicating an increased stress resistance in inoculated kernel. Compared to the indirect kernel-fungus interaction and to non-treated kernels (control), direct interaction produced highest effect on lipids. Among treatments, the fingerprint region (1800–800 cm^−1^) and SEM images indicated an important shift in glucose oligosaccharides, possibly linked to coleorhiza-polymer layer disappearance. Acquired differentiation in coleorhiza composition of *T. durum*, between ambient and drought conditions, suggests that FTIR spectroscopy could be a promising tool for studying endosymbiont-plant interactions within a changing environment.

## Introduction

Climate change is a key environmental stress for wheat (*Triticaceae*) in arid and semi-arid world regions^1^. Plant water-deficit is a vital driver of plant ecophysiology, phenophases, and reproduction efficacy, which affects seed and kernel germination as well as crop yield^2,3^. High temperature combined with drought is predicted to occur more frequently, with adverse effects on plant water economy, and is identified as a major cause of irreversible damage to plant physiology, function or development^4^. Phyto-beneficial microbes, particularly endophytes are partners of plants throughout its developmental stages, including germination, root and stem growth, and reproduction^5^. They improve water and nutrient uptake, as well as other signals important to plant development. Seed and kernel fungal endophytes have been classified as natural helpers involved in controlling germination under stress, and as crucial symbiotic moderators of biochemical and physiological systems providing prenatal care to plant^6^.

Several studies have suggested that the detrimental drought effect can be alleviated by improved mechanisms of adaptation^7^ or external acquisition of endosymbionts at the seed/kernel level^8^. Fungal endosymbionts enhance seed and kernel vitality (mycovitality) via biological stratification^9–11^, and act as plant growth promoters (PGPs) under climate/heat and drought stress conditions^6,12^. Studies on the symbiotic germination of wheat kernels provided molecular evidence of the importance of coleorhiza in improving germination via mechanism of biological stratification or control of hydrothermal time (HTT) of germination, in addition to reporting that the spatial distance between symbiotic partners may be a critical factor driving mycovitality^10,13^. These findings also shed light on coleorhiza and its regulation of phytohormonal function in wheat kernel-germinant. Although previously perceived as a mere protective layer^14^, coleorhiza is now known to regulate expression of gibberellins (GA) and abscisic acid (ABA) which improve kernel germination and vigor^10^.

Water-deficit stress poses a unique challenge to plant cells. According to Moore *et al*.^15^, drought tolerance involves growth adaptation to reduce water availability, and restructuring of the cell wall to allow growth processes at lower water contents. While desiccation tolerance is controlled by the evolutionary capacity of cell walls to resist against irreversible damage^16^, information lacks about the role of fungal endophytes in inducing coleorhiza adaptation, or molecular composition and polymer transformation, to cope with the water-deficit stress. Although it is well documented that adhesion of fungi to the plant root surface is essential to establish the symbiotic interaction^17^, no data exists about the adhesion, penetration, or initiation of the intimate kernel-fungus association via coleorhiza. Therefore, knowledge on the chemical components governing coleorhiza’s interaction with its fungal endosymbiotic partner under water scarcity merits exploration^6,10^.

Here, we hypothesized that wheat drought resistance (sustained or increased kernel vigor and germination rate) depends on coleorhiza-endophyte interaction mechanisms. We suspect symbiotic coleorhiza to protect wall integrity, thus keeping essential cell functions, water retention, and molecular profiles of lipids, amines, and carbohydrates in order to maintain the ability of kernels to germinate. In the last decades, spectroscopy techniques, involving Fourier transform infrared (FTIR) spectroscopy and hyperspectral imaging, were used for quick and reliable analysis of plant cell wall components, as well as of agricultural material such as grain, pollen, plants, forages and soils^18,19^. Despite its large potential in seed and kernel molecular biology and biochemistry, FTIR has not been applied to coleorhiza. It is also unknown whether distinct peaks amenable to spectral interpretation can be useful diagnostic tools in evaluating the effect of beneficial endophytes on coleorhiza drought tolerance^10^. Hence, we proposed the first-time use of FTIR spectroscopy, coupled with multivariate analysis, to depict the kernel-fungal endophyte-induced shift in coleorhiza’s chemical composition under ambient and drought stress *in vitro* conditions.

Our aim was to ascertain whether mid-IR spectroscopy could be employed to investigate biochemical changes in coleorhiza following kernel-endosymbiotic fungal treatment. FTIR spectroscopy was used to analyze coleorhiza’s ability to promote durum wheat (*Triticum durum* Desf.) kernel germination by evaluating its endosymbiont- dependent water retention and molecular profiles of lipids, amines and carbohydrates.

The ambient and drought stress conditions were studied and compared *in vitro*. FTIR spectroscopy was also used to further elucidate a correlation between the kernel-to- fungal endophyte distance on germination outcomes, a phenomenon responsible for biological stratification of kernels through shifts in phytohormonal pathways^10,13^. Scanning electron microscopy (SEM) was employed to assess anatomical changes of the embryo coleorhiza during hydration^20^ and breaking of kernel dormancy^14^. Thus, the goal was to prove the usefulness of FTIR spectroscopy^21,22^ as an alternative tool for characterizing the effect of endophytic fungus on nutrient and molecular changes in coleorhiza, which impact kernel germination and resistance to drought stress.

This study provides spectroscopic evidence that fungal endophytes and host-fungal partner distances play a role in wheat kernel germination - by modulating the expression of germination-related coleorhiza tissue structure, as well as by influencing water economy and the bioaccumulation of chemicals (e.g. nutrients and constitutive cellular coleorhiza molecules).

## Materials and Methods

### Plant and fungal materials

Plant fresh materials and kernels of AC Avonlea durum wheat (*Triticum durum*) were certified to be free of microbes. AC Avonlea possesses low resistance to a variety of environmental stresses including heat and drought^23^. The kernel of this highly-susceptible durum variety demonstrated an important fungal endophyte-driven improvement in resistance during germination and germinant establishment coping with heat and drought^6^. The kernels were surface sterilized, manipulated, and germinated as described elsewhere^10^. For this study, the endophytic fungal isolate *Penicillium* sp. SMCD 2206, from the Saskatchewan Microbial Collection and Database (SMCD), was used. Fungal cultures were grown at 21 °C in the dark and fresh cultures were used for *in vitro* assay.

### ***In vitro*wheat growth and sample preparation**

**Figure 1:**
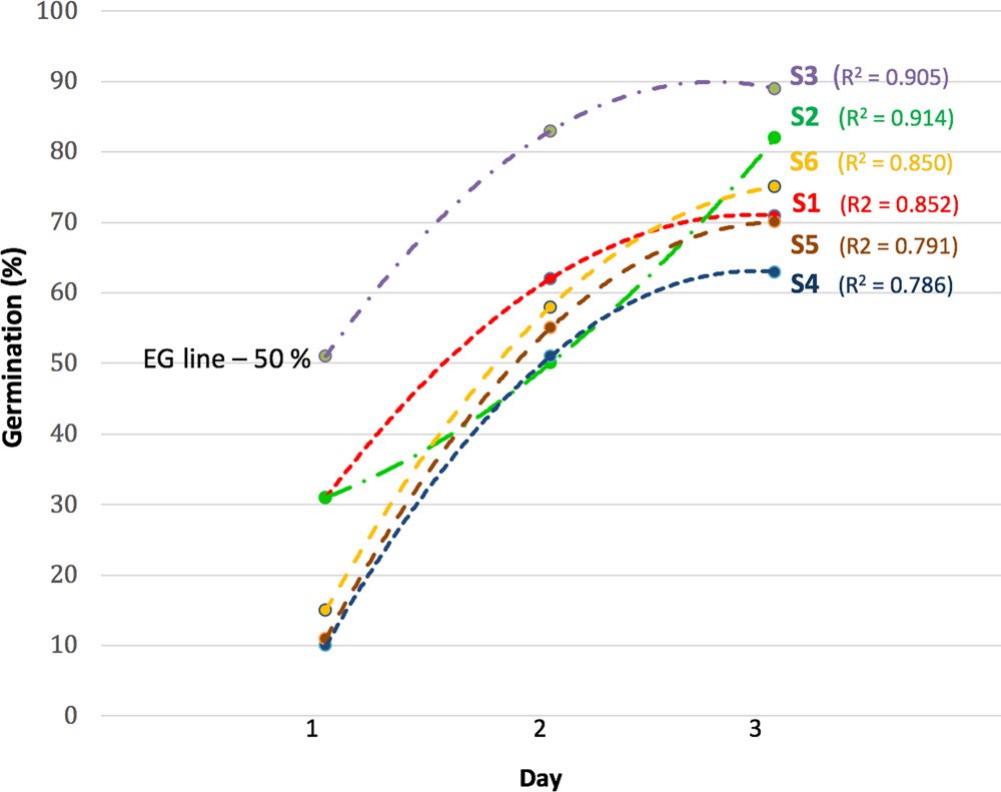
The level of wheat (*Triticum*) kernel biostratification induced by endophytic fungal SMCD 2206 inoculant *in vitro*. The kernels were exposed to S1–S6 treatments in reducing hydrothermal time (HTT) required to achieve 50% germination (EG-Energy of germination) combined with percent germination (24 h, 48 h and 76 h) attained and on day3 when the samples were taken for FTIR and SEM analyses. Ambient S1 (kernel germinated without SMCD 2206), S2 (kernel germinated apart from SMCD 2206) and S3 (kernel germinated in contact with SMCD 2206) grown on PDA. Drought S4 (kernel germinated without SMCD 2206), S5 (kernel germinated apart from SMCD 2206) and S6 (kernel germinated in contact with SMCD 2206) grown on 5% PEG.

**Figure 2:**
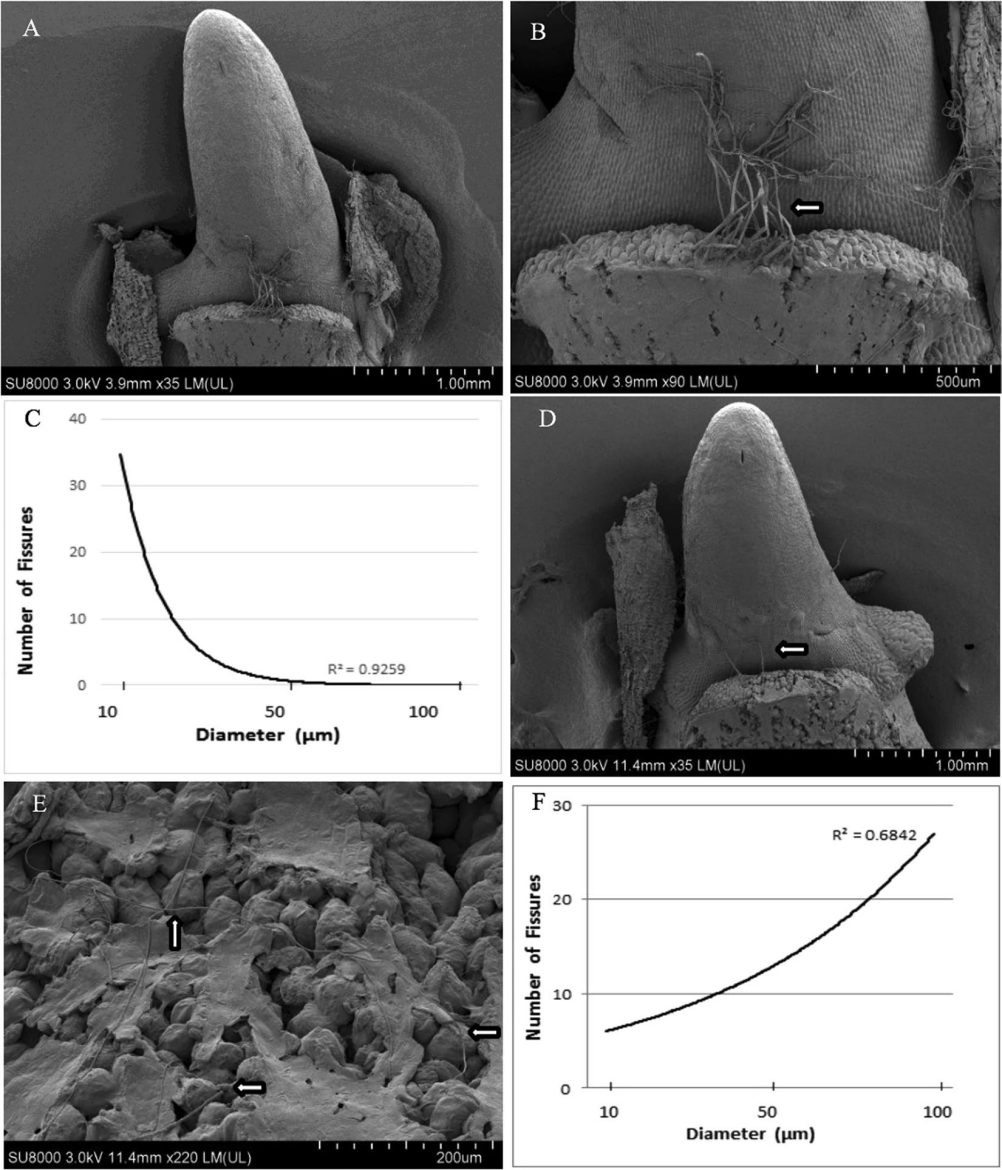
*In vitro* coleorhiza SEM analyses, root, and coleoptile growth of 3 days-old *T. durum*. Ambient condition: S2 - kernel germinated apart from SMCD 2206 (**A**–**C**) and S3 kernel germinated in contact with SMCD 2206 (arrows) (**D**–**F**) grown on PDA. The figure shows that coleorhiza’s polymer disappears more progressively showing larger fissures in S2 (**C**) compared to S3 (**F**) of *Triticum* germinating embryos in direct contact with SMCD 2206 (**E** arrows) as captured by SEM at 36 h of incubation. The fibrous filaments or coleorhiza-radicle joints break more slowly in S2 compared to S3 - as depicted by (**B**) and (**D**), respectively. Scale bars: (**A**,**D**), 1000 μm; (**B**) 500 μm; (**E**), 200 μm.

*In vitro* kernel germination (Fig. 1) and early coleorhiza activation (Fig. 2) were examined with and without *Penicillium* sp. SMCD 2206, and under ambient (PDA, potato dextrose agar) and drought (PEG, 5% polyethylene glycol [H(O-CH_2_-CH_2_)_n_-OH] amended to PDA) conditions^12^ to test the effects of fungal endophyte on shifts in molecular profile of the coleorhiza - driven by biostratification in germinating kernels^10^.

Fungal SMCD 2206 endophyte was grown on potato dextrose agar (PDA) at room temperature in darkness for at least three days before use. Wheat kernel germination was induced on the SMCD 2206 pre-inoculated agar plates^10^. After 1 day incubation of SMCD 2206, five surface sterilized kernels of AC Avonlea were inoculated on each prepared plate.

The indirect fungal effect was assessed according to Banerjee *et al*.^13^ with some modifications. An agar plug (5 mm^2^) of the endophyte dissected from the margins of the 3 day old-parent colony was placed in the center of a 90 mm plate with 1.5% PDA and 5% PEG, ensuring an indirect contact *via* exchange of volatiles. Then, 5 surface sterilized kernels were placed on the plate encircling the fungal agar plug at approximately 2 cm distance. All plates were sealed with 6 layers of Parafilm^®^ (Pechiny Plastic Packaging, Menasha, WI) to avoid diffusion of volatile/gaseous compounds. The impact of direct-contact of the fungal endophyte was observed by placing a 5 mm^2^ in the center of the PDA plates encircling the fungal agar plug at approximately 0.5 cm distance. All treatments were carried out with three replicates of PDA plates, with 5 surface sterilized wheat kernels on each plate. As control, five surface sterilized kernels were incubated without SMCD 2206. All plates were incubated at 21 °C in darkness. Wheat kernels were observed for germination rate before isolating coleorhiza at the growing points over three days (24 h, 48 h and 72 h) following the method described by Vujanovic *et al*.^10^. One repetition of germination experiment was performed, because our previous SMCD 2206-*Triticum durum* kernel studies *in vitro* demonstrated a consistent plant-fungus interaction^10,13^ achieving predictable, repeatable patterns of kernel germination response-driven by coleorhiza physiological/phytohormonal activation^13^. Combinations of coleorhiza samples collected from AC Avonlea germinating kernels on day 3 were as follows:

***Ambient-standard conditions***

**S1 -** Kernel incubated on PDA - Without SMCD 2206

**S2 -** Kernel incubated on PDA - Apart from SMCD 2206

**S3 -** Kernel incubated on PDA - Contact with SMCD 2206

***Drought-stress conditions***

**S4 -** Kernel incubated on 5% PEG - Without SMCD 2206

**S5 -** Kernel incubated on 5% PEG - Apart from SMCD 2206

**S6 -** Kernel incubated on 5% PEG - Contact with SMCD 2206

To investigate the change in composition of wheat coleorhiza, 3 days-old-kernel coleorhizae were gently collected by using tweezers under the Carl Zeiss magnifier^13^. On day 3, all treatments including control kernels reached a desirable 50% germination, which corresponds to energy of germination (EG) state^10^.

Germination rates (%) were calculated as the number of germinated kernels out of 90 kernels (5 kernels × 6 treatments × 3 replicates) that were planted.

Germination energy is the percent, by number, of kernels which germinate up to the time of peak germination, generally taken at 24 h, 48 h and 72 h period. It was calculated as follows: *Germination energy* = (cumulative germination percentage/days since sowing date) × 100%. In our experiments, 50% germination was associated to high germination energy^10^.

### SEM imaging

Scanning electron microscopy (SEM) images (Fig. 2) were obtained on two sets of germinating wheat kernels, under indirect (S2) and direct contact with endophytic *Penicillium* SMCD 2206 strain (S3), at day 3 using an Ultra-High Resolution (1.0 nm) Scanning Electron Microscope (Hitachi SU-8010 FE-SEM) operating at 3 kV. The germinating wheat kernels showing developed coleorhiza were dissected and then fixed with 2% glutaraldehyde (GA) in 0.1 M sodium cacodylate (NaCAC), at pH 7.2 for 3 hours at room temperature and then stored overnight at 4 **°**C. Then, the samples were washed with 0.1 M NaCAC three times and stored at 4 **°**C before osmium fixation. The pre-fixed samples were further fixed in 1% osmium in 0.1 M NaCAC for 1 hr and three times rinsed with 0.1 M NaCAC and sterile distilled water^24^. The fixed samples were dehydrated according to Fischer *et al*.^25^ with slight modifications using ethanol gradually (30%, 50%, 70%, 80%, 90%, 95%, and absolute ethanol) for 15 min. The last step was repeated three times. The dehydrated samples were placed on a critical point dryer (Polaron E3000). Afterward, the samples were coated with gold (10 nm thickness) using a Q150T ES Quorum, turbo-pumped sputter coater prior to examination. The developmental-opened fissures on a polymer layer covering coleorhiza-detected by SEM were assessed using three replicates of S2 and S3 samples under a Carl Zeiss Axioskop2 with a Carl Zeiss AxioCamICc1 camera^26^.

### FTIR spectra

Coleorhiza samples were collected by sterile Fisher brand razor blades. To remove any residual moisture, coleorhizae were lyophilized overnight in the freeze dryer prior to samples preparation for FTIR spectroscopy. Dried samples were then grinded by mortar and pestle to provide a homogeneous mixture. To prepare a potassium bromide (KBr) pellet or disk, each sample was added to a concentration rate of 1.3%, grinded with a mortar and pestle to a fine powder. The fine powder of sample/KBr mixture was transferred on the dye set and made into a pellet in triplicate under 7 psi (pound-force per square inch) for 2 minutes, using a hydraulic press (Manual hydraulic press 15 Ton, Specac, Orpington, UK). Pellets (12 mm) were transferred on clean sample dishes for FTIR measurement^22^.

FTIR spectra were collected using the Bruker IFS 66 V/S spectrometer with a KBr beam splitter at the mid infrared beamline using globar (silicon carbide) as the infrared source. Each IR spectrum was measured and recorded at resolution of 2 cm^−1^ and at sample scan time of 64 in the range of 4000–600 cm^−1^ ^22^. The same sample was repeatedly scanned three times for spectral data collection.

### Data analyses

A second derivative was applied to the spectra to compare the tripartite kernel fungal endophyte environment interaction. It allows evaluation of changes (increase/decrease) in each peak height as affected by the environment^19,22^.

**Figure 3:**
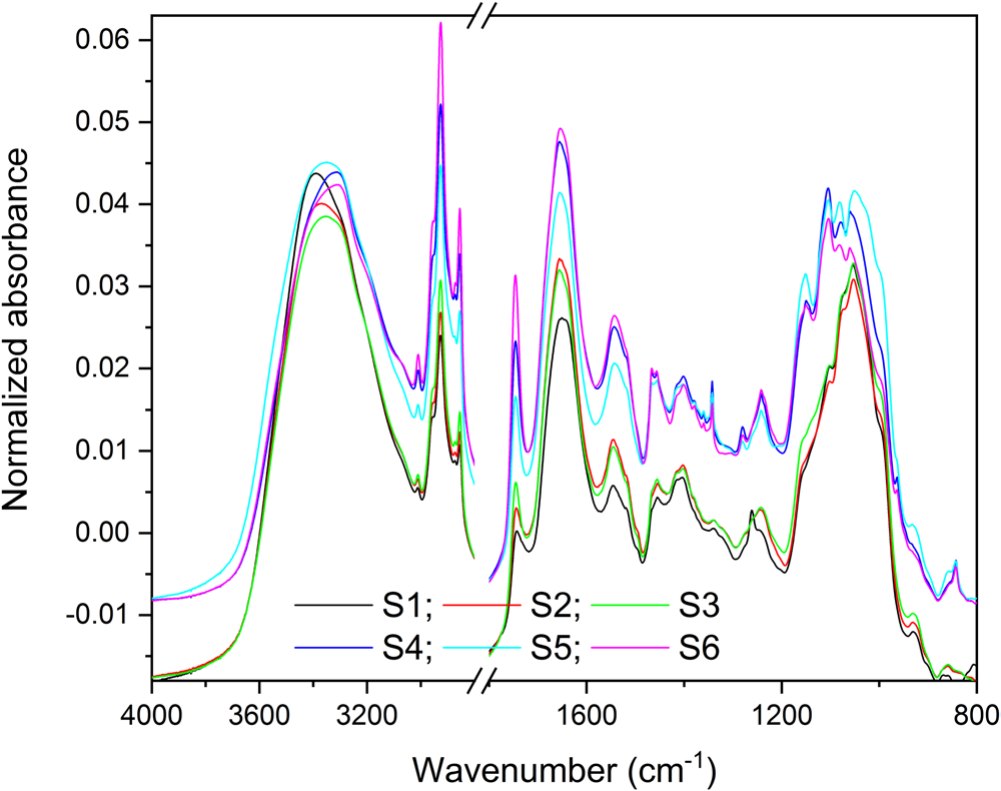
Vector normalized and average FTIR spectra of six treatments S1–S6.

**Figure 4:**
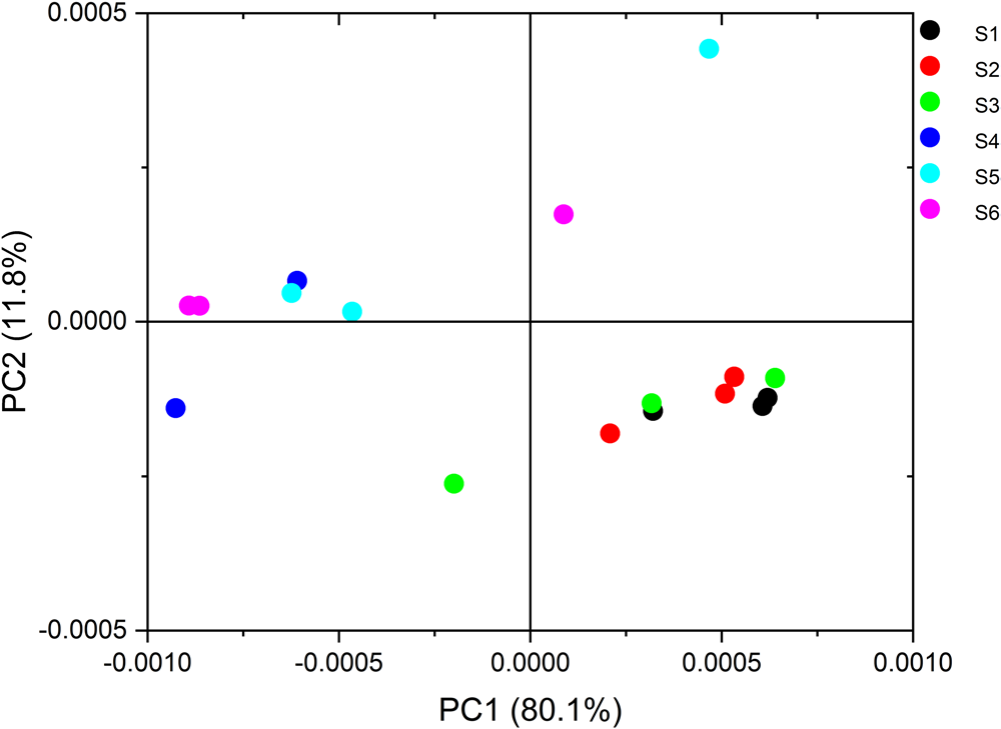
Scatter score plot based on principal component analysis (PCA) of FTIR spectra of coleorhiza.

The FTIR data analysis and plotting were carried out using OPUS (version 7.2, Bruker Optik GmbH) and Origin Pro (OriginPro 2018, OriginLab Corporation) softwares. For Fig. 3, the FTIR spectra were first baseline corrected using rubberband correction algorithm (64 points), vector normalized, and averaged using the OPUS software. For the PCA (Fig. 4 and Suppl. Fig. 2), the spectra were first baseline corrected and vector normalized. OriginPro software was used to generate the second derivatives spectra that were then used for the PCA analysis. The loading plots of PCA indicate which peaks/components are contributing more to the variation between treatments as determined by PCA axes. The major peaks of PCA loading plots were determined using the Quick Peaks routine in OriginPro with the settings of local maximum at 0% threshold height, no baseline, and area at *Y* = 0 (version 9.1, OriginLab Corporation, Northampton, MA, USA)^25^.

Assignment of the labeled bands representing different functional groups of compounds were analyzed and compared to the reference data published elsewhere^18,27–29^.

## Results and Discussion

### Germination

The coleorhiza-endophyte interaction mechanism proved to be efficient in maintaining kernel vigor, as well as in increasing kernel germination rate (%/time) and energy of germination (EG) (Fig. 1). Symbiotic coleorhiza kept cell functions and reduced HTT-hydrotermal time by increasing ability of kernels to germinate under drought stress.

To determine if our combined drought and endophyte stratification treatments were superior compared to standard treatments, we measured the germination rate and efficacy and compared them to control groups.

In ambient conditions, S3 (89% - kernel germinated in contact with SMCD 2206) and S2 (83% - kernel germinated apart from SMCD 2206) were more performant than ambient S1-control (72% - kernel germinated without SMCD 2206). Germination rates under drought, S6 (75%- kernel germinated in contact with SMCD 2206) and S5 (70% - kernel germinated apart from SMCD 2206) were above S4 (64% - kernel germinated without SMCD 2206). Both kernel-endophyte direct and indirect (apart position) interactions triggered stratification by increasing germination values above that of the control group by the end of the day 3. Only S3 stratification or direct kernel -SMCD 2206 contact under ambient condition reached EG-50% germinated kernel level on day 1, thus showing the highest increase in germination energy at day 1 (Fig. 1).

In all treatments (S1–S6), maximum slope of the curves was always between day 1 and day 2, while gradual increase in germination energy was detected between day 2 and day 3.

During the entire 3-day experiment under ambient and drought conditions, kernels in direct and indirect contact with endophyte showed the best germination rate. This data corroborates previous findings about the kernel-SMCD 2206 stratification or capacity to enhance kernel germination rate and vigor^10^.

### SEM imaging

Coleorhiza is a non-vascularized multicellular embryonic tissue covering the seminal roots of Poaceae (e.g. *Brachypodium*, *Oryza*, *Hordeum*, *Triticum*) kernels. Coleorhiza is a sheath-like structure thought to have a role in protecting the emerging root^30^. Recently, it has been associated with the regulation of breaking dormancy and improved germination in *Triticum* through a delicate balance between biosynthesis of gibberellins (GAs) and degradation of abscisic acid (ABA); it appears to be critical for modulating the GAs/ABA concentration level ratio in coleorhiza^10,13^.

In this study, coleorhiza structural differences were observed between S3 germinating kernel in direct contact with SMCD 2206 and S2 germinating kernel apart from SMCD 2206 (Fig. 2): the two treatments were associated to the most dramatic promotion of germination at day 3 compared to other treatments under ambient conditions (Fig. 1). During S2 and S3 germination, coleorhiza appeared as the first structure protruding after pericarp and testa rupture (coleorhiza emergence), followed by coleorhiza rupture to allow root emergence - indicating the end of germination *sensu stricto* (Fig. 2A,D), which concord with data for *Brachipodium* and *Hordeum*^14,31^.

During the development, coleorhiza undergoes changes and appears as independent structure covered by a polymer layer possibly reach in mannan or mannose^32^. As this structure approaches maturity, a combined cell-expansion pressure and degradation is exerted on the polymer layer. As presented in Fig. 2, S2 coleorhiza apart from SMCD 2206 experienced a more gradual “disappearance” of the polymer layer, with the highest frequency of 10–30 µm fissures/openings (Fig. 2B) after 72 h imbibition. In comparison, the S3 coleorhiza polymer layer in direct contact with SMCD 2206 showed a higher frequency of 50–100 µm fissures/openings (Fig. 2E) after 72 h imbibition. The assessed morphometric analyses of fissures measured through SEM show a dramatic coleorhiza system advancement induced by the fungal endosymbiont (Fig. 2E, arrows).

The phyto-beneficial *Penicillium* strain used in this study showed adaptability to coleorhiza’s topography by producing multiple fungal structures to establish an intimate fungus-host relation (Suppl. 1). During a direct SMCD 2206-coleorhiza surface contact, the fungus formed typical *rhizomorphs* (Fr+) or “root-forms” of hyphal aggregation. These threadlike or cordlike structures are made up of parallel hyphae with similar functions to plant roots; they are capable of conducting nutrients over long distances and supply necessary water and minerals to plant. The fungal appressoria, or special hyphal pressing organs, on the polymer surface as well as typical penetration hypha, needed to break down the polymer layer and infect coleorhiza’s internal tissue, are formed. These fungal vegetative structures were associated with the formation of large polymer openings occupied by hyphae, or branching fungal filaments, responsible for endosymbiotic colonization of the coleorhizal tissue. While the topography of the colonized (Fr+) polymer surface progressively changed towards large fissures/openings, the non-colonized (Fr−) polymer showed only small openings on the surface of coleorhiza.

González-Calle *et al*.^32^ suggest that the enlarged fissures are possibly associated with disappearance of mannan polymers due to hydrolysis of catalyzed by endo-*β*-mannanases and endo-*β*-1,3-glucanases. The genes encoding hydrolytic enzymes involved in the degradation of these polymers were associated with rice (*Oryza*) postgermination events (~3 days of imbibition) and with elongation of barley (*Hordeum*) as well as purple false brome (*Brachypodium*) coleoptiles^32–34^. The described phenomenon seems to represent an important phase in *Triticum* germinating kernels, since polymer disappearance increased as germination progressed. Hence, there may be a connection between endophyte-driven stratification (bio-stratification) and mannan polymer disappearance; symbiotic germination may provide an additional energy (mono- and oligosaccharides) supply. There is a body of evidence on plant^32^ and fungal endo-*β*-mannanases (MAN) producers including *Penicillium* and *Aspergillus*^35,36^. To the best of our knowledge, this study is the first suggesting that kernel endophytic fungus (*Penicillium* sp. SMCD 2206) activity may potentially alter and/or accelerate polymer disappearance or presumably mannan-structure hydrolysis related to fungal MAN accumulation in coleorhiza^35,36^. Although the MAN gene and amino acid sequences have been elucidated in plant monocots, namely *Hordeum*, *Brachypodium* and *Oryza*, *Triticum* (wheat) MAN amino acid sequence was not reported. Based on coleorhiza BdMAN1DNA sequence retrieved from González-Calle *et al*.^32^ and blasted against NCBI public database, it appears that mannan endo-1,4-beta-mannosidase 1 (LOC100846205) originating from *Brachypodium distachyon* is similar to *Oryza sativa* var. *japonica* mannan endo-1,4-beta-mannosidase 1 (LOC4327860). Using the same approach, the BdMAN1sequence was blasted against SwissProt –Expasy database (https://web.expasy.org/tmp/1week/blastf66305.html#Al19) on 12/05/2018. The Expasy blast resulted in A0A1D5WIE7 protein sequence being identified as uncharacterized protein OS = *Triticum aestivum*. This sequence blasted against NCBI showed 86% similarity to XP_015689492 mannan endo-1,4-beta-mannosidase 1 in *Oryza brachyantha* [length: 432] and 72% similarity to XP_003569504 mannan endo-1,4-beta-mannosidase 1 in *Brachypodium distachyon* [length: 417]. This is the first attempt to relate the coleorhiza mannan-polymer layer disappearance process across symbiotic *Penicillium-Triticum* kernel germination and merits further investigation.

### FTIR spectra

#### Unraveling the biochemical changes in the coleorhizae composition

Baseline corrected, FTIR spectra of the coleorhiza composition of *Triticum* in the 4000–800 cm^−1^ regions are seen in Fig. 3. *Triticum* samples showed substantial differences between ambient (PDA) and drought (5% PEG) conditions. The broad and very strong band at 3375 cm^−1^ was assigned to various compounds, which seem to be mainly N-H stretching of proteins with some contribution from O-H stretching of polysaccharides and intermolecular H bonding. It is suggested that the OH-stretch frequency is sensitive to the bond’s local environment, structure and dynamics of water^35^. S1 ambient-control peak is lower than S2 and S3 (kernel-fungal endophyte) treatments. Inversely, S4 drought-control’s highest peak is considerably above S5 and S6 (kernel-fungal endophyte) treatments.

Band assignations are as follow: acyl lipids chain at 3008 cm^−1^ olefinic=CH; asymmetric and symmetric CH_2_ stretching of mainly lipids at 2926 cm^−1^ and 2854 cm^−1^; CH_2_ bending of mainly lipids at 1462 cm^−1^; and ester C=O stretching of triacyl glycerols at 1747 cm^−1^. FTIR results showed higher band absorption at 1720–1740 cm^−1^ (Fig. 3) for non-inoculated controls (S4) samples relative to that of inoculated (S5 and S6). This is indicative of reduced oxidative stress/reactive oxygen species (ROS) associated with symbiotic kernel, or the possible regulation of phenylpropanoid pathway^22^.

Bands within the region between 1540 and 1642 were assigned to proteins. In the protein region, the two strong bands were associated to Amide I at (1652 cm^−1^) and Amide II (1546 cm^−1^). The carbohydrate bands were depicted with C-O stretching vibrations at 1152 cm^−1^ and 1054 cm^−1^. According to Lahlali *et al*.^22^, the protein Amide I region, especially L-phenylalanine at 1652 cm^−1^, can be implicated in plant resistance responses to stress via phenylpropanoid and lignin.

The second derivative spectra Suppl. Fig. 2 in the lipid region (3100–2800 cm^−1^) of S1 to S6 showed a difference between PDA and 5% PEG. Two strong peaks at 2926 cm^−1^ (asymmetrical CH_2_) and 2854 cm^−1^ (symmetrical CH_2_) in the secondary derivative spectra help distinguish different media condition. Increase in lipid regions of S4, S5, and S6 (5% PEG) compared with S1, S2, and S3 (PDA condition) may imply that a drought condition stimulates more lipid production in coleorhizae. Furthermore, it appears that contact or direct interaction between the fungus and the kernels contribute to the increase of lipids content in coleorhizae.

The second derivative of absorption spectra in the fingerprint region (1800–800 cm^−1^) depicted a strong difference between PDA and 5% PEG condition. Three relatively strong peaks were observed at 1747 cm^−1^ ester C=O (Suppl. Fig. 2) stretching of triacylglycerols or pectin, at 1462 cm^−1^ CH_2_ bending of mainly lipids or cellulose, and at 1348 cm^−1^ CH_2_ bending of mainly lipids with some contribution from proteins or cellulose and pectin^37^. Weak lignin (1525–1505 cm^−1^), ribose (1150 cm^−1^) and starch (1124 cm^−1^) bands were also depicted by the spectral signatures. However, some more pronounced carbohydrates bands were detected at 1017 cm^−1^, 990 cm^−1^ and 861 cm^−1^, corresponding to pectin, hemicellulose (arabinoxylans), and starch, respectively^18^.

#### Principle Component Analysis (PCA)

Qualitative PDA–PEG spectra differences were depicted using normalized two-dimensional spectra (Fig. 4). Data values are clearly separate S1–S3 from S4–S6 data sets. There is overlap between some individual groups due to variability among replicates. As shown in Fig. 4, the scatter plot of PC1 and PC2 explained 91.9% of variability among the coleorhizae under different media conditions. The PC1 axis mostly distinguished S1, S2, and S3 (exposed on PDA condition) on the positive side of PC1 from S4, S5, and S6 (drought- 5% PEG condition) which was scattered on the PC1. S1, S2, and S3 (ambient - PDA condition) clustered on the negative side of PC2, whereas S4, S5, and S6 (exposed on 5% PEG condition) were spread along the PC2 axis.

As shown in Suppl. Fig. 2, the positive influence of PC1 loadings had two peaks at 2926 and 2854 cm^−1^ (asymmetrical and symmetrical CH_2_ stretching), whereas the negative influence of PC1 had two peaks at 2945 and 2839 cm^−1^. The influence of PC2 loading was not significant. Therefore, PC1 showed that the lipid composition of coleorhizae changed among treatments.

As shown in Suppl. Fig. 2., the positive influence of PC2 loading was shown at 1136 cm^−1^ (O-C-O asymmetric stretching) and 1070 cm^−1^ (C-O stretching and C-C stretching), potentially indicating carbohydrates such as D-glucose oligosaccharides represented by *β*-1,4-mannobiose, *β*-1,4-manotriose^38^, linked to D-mannose residues; and possibly glycosidic bonds and cyclic structures of monosaccharides^39^. On the other hand, the negative influence had peaks at 1152 cm^−1^, 1109 cm^−1^ (C-O stretching and C-C stretching), and 844 cm^−1^ (Ring vibration), indicating carbohydrates which might include mannan. PC1 loadings were not significant. Therefore, it seems that the content of carbohydrates changed among treatments. It would not be surprising for mannobiose to likely accumulate in hydrolytic activities of *Penicillium* SMCD 2206. Fungi produce *β*-mannanases to hydrolyze mannan yielding mainly mannobiose and mannotriose^40^. Mannanase enzymes production by several hemicellulolytic fungi such as *Penicillium purpurogenum*, *Trichoderma harzianum*, *Polyporus versicolor*, *Thielaviaterrestris*, *Aspergillus tamarii* and *Aspergillus niger* has already been reported^41^.

In conclusion, this study is the first report highlighting the use of FTIR spectroscopy to understand early plant-endophyte interaction based on molecular changes in coleorhiza during kernel germination stage. It demonstrates that the chemical composition of coleorhizae markedly changed under different situations of drought stress. A variety of functional groups seem to contribute to observed chemical differences, including O-H stretching, acyl lipids chains, proteins, polysaccharides carbohydrates, hemicelluloses, and possibly mannan and glucan. SEM microscopy was useful to differentiate coleorhiza nonvascular tissue, and to potentially begin bridging the mannan-polymer layer rupture hypothesis in relation to coleorhiza direct vs. indirect contact with the endosymbiont.

## Electronic supplementary material

Supplementary Information

## Data Availability

All data generated or analyzed during this study are included in this published article.
