# Supplementary material for: Spectroscopy and SEM imaging reveal endosymbiont-dependent components changes in germinating kernel through direct and indirect coleorhiza-fungus interactions under stress
Source: Sci Rep. 2019 Feb 7;9:1665. doi: 10.1038/s41598-018-36621-8 (PMC6367502; doi:10.1038/s41598-018-36621-8)
Supplement: Supplementary file 1 — Supplementary Information [file 41598_2018_36621_MOESM1_ESM.pdf]

Supplementary Information

Article in Scientific Reports

Spectroscopy and SEM imaging reveal endosymbiont-dependent components changes in germinating kernel through direct and indirect coleorhiza-fungus interactions under stress

Vladimir Vujanovic, Seon Hwa Kim, Rachid Lahlali, Chithra Karunakaran

Supplementary figures S1

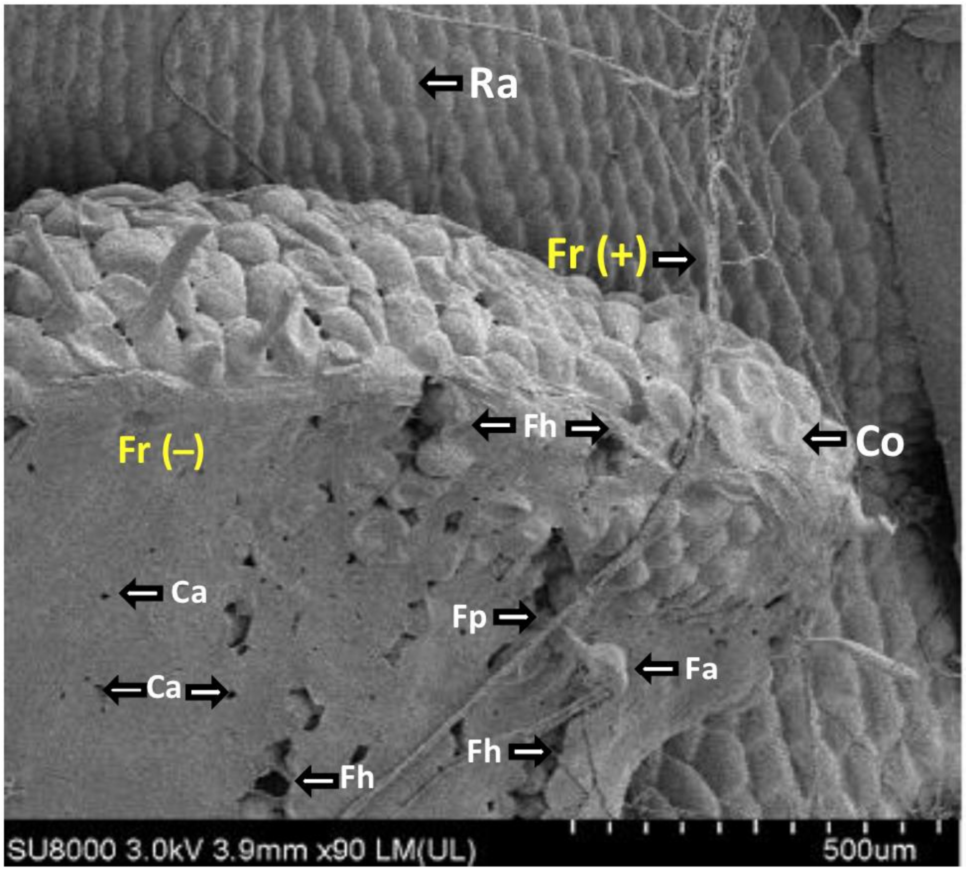

Supplementary Figure 1. Comparison between SMCD 2206 colonized (Fr +) vs. non-colonized (Fr -) coleorhiza zones. Ra: radicle (plant embryonic root), Co: coleorhiza, Ca: coleorhiza aerial small (Fr **+**) openings, Fr: fungal *rhizomorph* or "root-form" hyphal aggregation, Fa: fungal appressorium or special hyphal pressing organ, Fp: fungal penetration hypha specific for infection, Fh: fungal colonization hyphae within large openings associated with direct *Penicillium*-polymer (Fr +) contact zone.

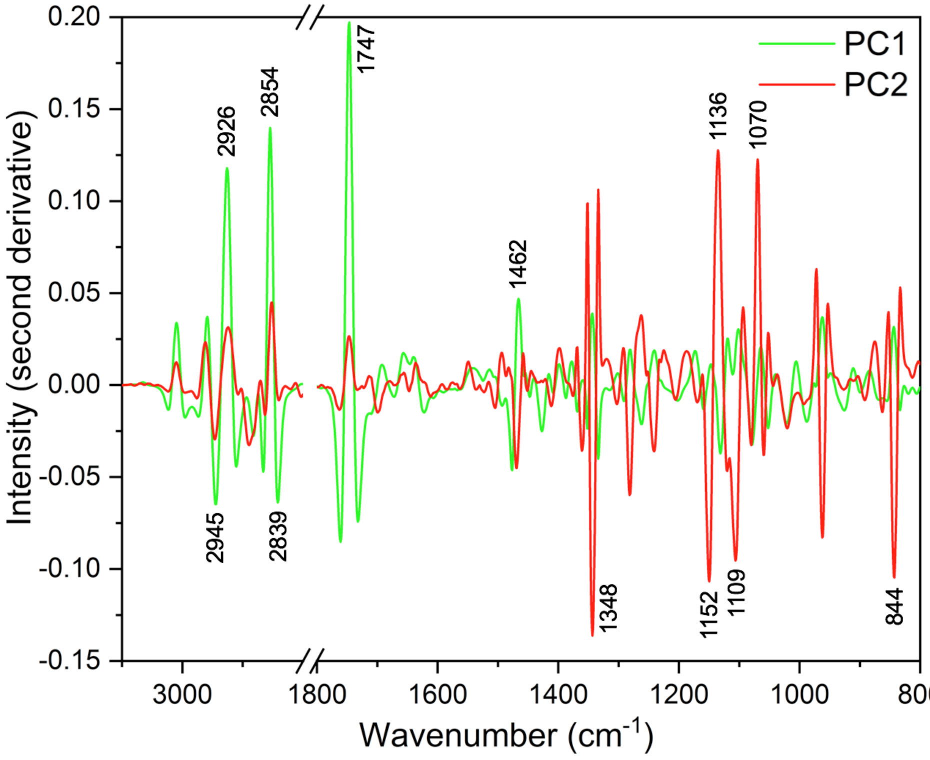

Supplementary Figure 2. PCA analysis – loadings plot.
